# Effect of a High Welfare Floor and a Concrete Slatted Floor on the Growth Performance, Behavior and Cleanliness of Charolais and Limousin Heifers: A Case Study

**DOI:** 10.3390/ani12070859

**Published:** 2022-03-29

**Authors:** Jakob Leskovec, Mojca Voljč, Silvester Žgur

**Affiliations:** Department of Animal Science, Biotechnical Faculty, University of Ljubljana, Groblje 3, 1230 Domžale, Slovenia; mojca.voljc@bf.uni-lj.si (M.V.); silvo.zgur@bf.uni-lj.si (S.Ž.)

**Keywords:** high welfare floor, beef heifers, behavior, cleanliness, Limousin, Charolais

## Abstract

**Simple Summary:**

The barn floor needs to provide a confident and comfortable walking surface while retaining durability and affordability. The floor of the barn is also important in order to achieve the best performance, health and welfare of the animals. Therefore, we compared a high welfare floor (HWF), which should offer higher comfort, to a concrete slatted floor (CSF), which is a standard housing system for cattle rearing. In the trials, Charolais and Limousin heifers were used testing the latter systems. We observed that heifers housed on an HWF tended to exhibit more species-specific behaviors, namely rubbing, grooming and aggression, and seemed to be cleaner than those housed on a CSF.

**Abstract:**

Various floor systems are used in cattle housing with different characteristics in terms of roughness, abrasion, wetness, bedding material, ease of cleaning, etc. Thus, the activity and welfare of the animals are greatly influenced by the type of floor. The floor of the barn can influence the development of health diseases, technopathies and the production and quality of animal products. Therefore, in the present case study, we studied the effects of two different flooring systems on the performance and on some behavioral and cleanliness parameters in heifers. Two floor systems (concrete slatted flooring (CSF) and high welfare flooring (HWF)) and two breeds (Charolais and Limousin) were used in the experiment. Heifers on HWF tended to show a higher frequency of grooming, rubbing and aggression than those on CSF, but not of standing, lying, eating, drinking, rumination, resting, stereotypies and covering of the animals. In addition, animals housed on HWF also appeared to show higher cleanliness than those housed on CSF. Results indicated that animals housed on HWF exhibited more social and self-care behaviors, suggesting that animals housed on such floors show more species-specific behaviors and have higher welfare.

## 1. Introduction

Cattle play a crucial role in providing food, especially protein, for humans, and also contribute to the preservation of the cultural landscape [1]. However, even though cattle farming has some clear advantages, it is doomed to produce many soil, water and air pollutants, especially ammonia and greenhouse gases [2]. Therefore, many attempts have been made to reduce the production and management of these pollutants. These mainly include changes in nutrition, barn design, flooring and bedding, and changes in manure management [3,4]. Another important key factor in cattle production is the design of production systems that meet the behavioral and physiological needs of cattle, thus improving production and quality parameters, animal activity and animal welfare [5]. Improving the welfare and health of cattle is under constant scrutiny by the public, who demand improvements. Farmers and policy makers must therefore meet this demand with educational and technological measures [6].

In the past, most of the research and practical improvements in barns have been made with regard to animal performance and working efficiency. The greatest improvement in this regard has been the change from tie-stall barns to free-range housing. In the last years, housing systems have been majorly improved. However, behavior and welfare, control of the microclimatic conditions, ammonia and greenhouse gas emissions, and manure management will be emphasized in the future [5].

In addition to the lower environmental impact of the barns, a great deal of emphasis is placed on the welfare of the animals. Studies show that comfortable bedding and free movement of animals are very beneficial to animal welfare [5,7]. In recent decades, fully slatted floors have become very popular, but cattle kept on slatted floors are more likely to suffer various injuries, especially of legs and claws [5,7,8], than animals kept on more comfortable floors. The locomotion of cattle is also strongly influenced by the type of floor, and especially by its coefficient of friction. A slippery floor on slatted concrete causes shorter strides and slower speed than solid concrete or a continuous rubber floor. Moreover, sand improves steps and speed compared to a continuous rubber floor and a solid concrete floor [9]. Researchers also came to similar conclusions in finishing cattle, where young bulls reared on perforated concrete flooring had a higher number of slips and a longer lying time, with a lower number of lying/standing transitions and longer lying duration than bulls reared on a rubber mattress [8]. A slippery floor on slatted concrete causes shorter strides and slower speed than solid concrete or a continuous rubber floor. Moreover, sand improves steps and speed compared to a continuous rubber floor and a solid concrete floor [8]. Researchers also came to similar conclusions in finishing cattle, where young bulls reared on perforated concrete flooring had a higher number of slips and showed more lying trials, with a lower number of lying/standing transitions and longer lying duration, than bulls reared on a rubber mattress [8]. In the experiment with fattening bulls reared on a slatted floor, bulls had less lying time and a higher number of skin lesions compared to a slatted floor covered with rubber [10]. Differences in lying times, hairless areas and other musculoskeletal pathologies/lameness were found in finishing beef cattle housed on deep litter in favor of fully slatted concrete floors [11]. The type of floor is also directly related to the cleanliness of the cattle, as shown in a study comparing a fully slatted floor, a fully slatted floor covered with perforated rubber mats and a straw bedding, with the animals housed on straw (6.12 kg/animal per day) being the cleanest [12].

To reduce ammonia emissions and improve animal welfare, the high welfare floor (HWF) was developed. This floor is made with special panels with a plastic comb, covered with comfortable foam and wrapped with semi-permeable foil. This allows urine to drain away very quickly while retaining fecal matter on the surface, which is removed manually or with a specialized robot. Therefore, ammonia emissions are reduced while a firm and comfortable floor is provided for the cattle. To the best of our knowledge, there have been some trials with dairy cows [5] but none with beef cattle, especially heifers. Therefore, we conducted a trial with Charolais and Limousin heifers housed on HWF or a concrete slatted floor (CSF) to evaluate growth, some basic behavioral traits and cleanliness of the animals.

## 2. Materials and Methods

### 2.1. Animals and Experimental Design

All the procedures with animals were performed in accordance with the legislation of Slovenia (UL RS nr. 32/21), which is harmonized with European legislation (Directive 2010/63/EU), for which no ethical committee approval is required.

The case study was carried out at the Educational and Research Animal Husbandry of the Department of Animal Science, Faculty of Biotechnology, University of Ljubljana, Slovenia. Fourteen Limousin (LIM; average age 258 days; body weight 311 kg) and 14 Charolais heifers (CHA; average age 254 days; body weight 343 kg) were included in the study. Animals were divided in two groups within the breed, with similar average weight and variability. The trial lasted from 21 November 2020 to 30 April 2021. The animals were housed in a closed barn with several pens. Animals were divided into four groups (7 animals per group) according to breed and flooring system. In the experiment, we had two different flooring systems, CSF and HWF (ID Agro, Lamelerveld, The Netherlands). The concrete slatted floor had 14 cm solid parts and 3.2 cm slots, while HWF was composed of plastic comb, foam mattress permeable to urine and altogether covered with semi-permeable foil. Urine drained under the floor, while the solid parts were removed manually once per day. Prior to the start of the study, the acclimation period lasted 42 days to allow the animals to become accustomed to both housing and diet. Individual animals were weighed every four weeks and the average per group was calculated (Table 1).

The animals were allocated to 4 pens of 7 heifers each, located in the same barn. The size and area of all four pens were the same. The size of each pen was 12 m × 4 m, and the area of each pen was 48 m^2^ (6.86 m^2^ head; space at the manger 1.7 m/head). Each pen was equipped with two drinkers so that the animals could drink water ad libitum. Pens with CSF were located on one side of the barn and pens with HWF were located on the other side of the barn, separated by the feeding area. Adjacent pens were separated by a metal bar.

### 2.2. Behavior and Cleanliness Evaluation

Four behavioral observation sessions were conducted during the experiment, with four weeks between observations. Direct observations were performed by three trained assessors using the scan sampling technique [13], with a 5 min interval between the scans. At each interval, the animal position (standing, lying) and activity (inactivity—standing or lying, feeding, rumination, stereotypy, grooming and rubbing) of each heifer were evaluated by the scan sampling technique [13]. Drinking, mounting and aggression were recorded continuously as events within each pen using the behavior sampling technique [13]. An aggression event was considered when the animal intentionally hit the other animal with the head. Each observation session lasted 8 h, from 8 am to 4 pm, starting immediately after the feed delivery. Fourteen days after the second observation, animals kept on HWF were moved to CSF and vice versa, in order to allow a crossover design of the trial.

The hygiene scoring system with minor adaptations (instead of the udder, the lower front limb was evaluated) was used to evaluate cleanliness [14]. The system was used to document the degree of manure contamination in 5 different areas using a 5-point scale (1—clean, without any manure, to 5—dirty, heavily covered with manure). The areas evaluated were classified as follows: tail head, upper rear limb, lower rear limb, ventral abdomen and lower front limb. The assessment was performed twice, 14 days after the second and after the fourth behavioral observation.

### 2.3. Diet and Chemical Analyses

Hay and water were offered to the animals ad libitum, while 1 kg of maize and soybean meal (50%:50%) were distributed in two equal daily amounts. Once a month, hay samples were taken for chemical analysis for the purpose of proximate analysis (AOAC, 2000). The proximate analysis (moisture, crude ash, crude protein, crude fat, crude fibre) of the feed was determined according to standard procedures (AOAC, 2000), dry matter (dried in the oven at 95–100 °C, AOAC method 934.01), crude protein (copper catalyst Kjeldahl method, AOAC method 984.13), crude fat (AOAC method 920.39), crude fibre (fritted glass crucible method, AOAC method 978.10) and crude ash (AOAC method 942.05). Hay had an average of 943.3 g/kg dry matter (DM), 139.1 g/kg DM crude protein, 20.8 g/kg DM crude fat, 261.3 g/kg DM crude fiber, 88.9 g/kg DM ash and 489.8 g/kg DM nitrogen-free extract.

### 2.4. Statistical Analyses

The Means procedure of SAS software was used for statistical analyses of the data (ver. 9.4, Sas Inc., Cary, NC, USA). The results of the behavior assessment were calculated as the sum of all the behavioral patterns per hour and expressed as a percentage.

## 3. Results

### 3.1. Performance Parameters

The animals of both breeds adapted well to the present housing systems. They grew as would be expected for heifers of this age and weight, although the Charolais animals had higher weight and weight gain than the Limousin heifers throughout the whole trial due to the breed specific differences. The flooring system did not seem to have an effect on the growth performance of the animals (Table 1).

### 3.2. Behaviour Parameters

Heifers in the present trial did not show behavioral abnormalities in the rearing systems, although there appeared to be some differences in observed behavioral parameters. Animals in the HWF group spent more time grooming (diff. 28.3%) and rubbing (diff. 62.8%) and showed more aggression events (diff. 99.0%) than animals in the CSF group. There were no differences observed between groups in the times of standing, lying, inactivity, eating, rumination, stereotypies and drinking (Table 2).

### 3.3. Cleanliness

The animals in the present trial appeared to be cleaner in the HWF group compared to the CSF group in all the observed body sites, except at the base of the tail. Heifers in group HWF were cleaner on the upper rear limb (−1.107 points), ventral abdomen (−0.929 points), lower front and rear limb (−0.714 points and −0.857 points, respectively) and joint score (−3.75 points) compared to those in group CSF. Cleanliness scores were also better for LIM in the ventral abdomen (−0.571 points), lower front limb (−0.929 points) and joint score (−2.04 points) compared to CHA (Table 3).

## 4. Discussion

To address the issues with welfare and ammonia emissions, HWF was developed. Although development is still ongoing, there are some evidences that HWF improves the performance of cows and some key behavioral indices of their welfare [5].

In the present trial, there appeared to be no meaningful differences in the performance of the animals. The latter is consistent with results reporting that the flooring system had no effect on the growth or weight gain of the cattle. In the experiment on finishing beef steers, authors did not find differences in the growth parameters between steers reared on either fully slatted concrete, slatted rubber mats or solid rubber mats [15]. Similar results were obtained in a study in finishing beef steers, in which fully slatted floors, slatted floors covered with rubber mats or solid floors bedded with straw had no effect on the performance parameters [12]. There were no differences in the growth and feed intake between beef bulls housed on a slatted floor or on a straw bedded floor [16]. On the other hand, fattening bulls on rubber-covered slatted floors had higher daily gains than bulls housed on concrete, for animals with similar weights to those in the present study [17]. Similar conclusions were drawn for dairy cows reared on compost bedding compared to loose earth flooring; cows reared on compost bedding had better conversion efficiency and milk production, which may be attributed to a longer lying of the cows.

In the trial we conducted, we had a low stocking density, so stocking density most likely had no effect on animal behavior since trials with cattle have shown that high stocking density, in particular, affects the laying, standing and feeding times of the animals [18,19]. In the present trial, heifers of the CHA breed achieved higher weights throughout the trial, with higher weight gains, regardless of the flooring system used. This is consistent with trials in which Charolais heifers achieved higher final body weights and weight gains under the same conditions as Limousin heifers [20]. This was also shown in fattening bulls, where Charolais had a higher final body weight than Limousin [17].

Awareness of cattle welfare on farms has grown significantly in recent decades. This has led to research, legislation and nonregulatory animal welfare assessment systems. [21]. It is difficult to define what is normal behavior for cattle, although pasture behavior should be considered normal behavior since this is the environment in which cattle evolved as a species [22]. Cattle engage in many behaviors while on pasture, the most common being standing grazing, standing resting, lying resting and walking, which together account for 96.6% of all observations of beef cattle on pasture [22]. Compared to our trial, the cattle on pasture were standing in about 85% of the scans [22]. In the present trial, it seems that the type of floor or breed had no effect on standing or lying time. This is in contrast to data in dairy cows, where animals housed on HWF had higher activity, measured as steps/h, compared to a slatted floor, even though the available lying area was very different among the systems [5]. The results also comply with trials on beef bulls comparing a slatted floor with a rubber-covered slatted floor [15,17] and deep litter [11,23], where the floor type had no effect on standing or lying. On the other hand, some trials have shown that solid rubber mats in finishing steers [15] and perforated floors coated with rubber mattresses in finishing bulls [8] increase lying time compared to slatted floors. In contrast to these results, bulls housed on a concrete floor, lie for longer than bulls housed on rubber floors [10]. Variations could be a consequence of pen space per animal, pen size, animal weight, number of animals in the pen, comfort of bedding or feeding management, as is the case with dairy cows [21]. In the present study, we had significantly more space per animal than recommended, so space per animal may be a less limiting factor.

In the present trial, it appears that neither the breed nor the floor type had any effect on the frequency of feeding, drinking and rumination. This is in agreement with the results of the experiment with cattle, in which the animals on concrete slatted floors did not feed for longer than on rubber-covered slatted floors [15,17] or deep litter [23]. On the other hand, trials in finishing bulls showed that straw bedding [16] and rubber coating on a slatted floor [8] led to a higher feeding frequency when compared to a slatted floor. The feeding frequency did not change in the trial with beef cattle on deep litter compared to a slatted floor [23], or on rubber mats compared to a slatted floor. The same applies to rumination, where, in beef cattle, the flooring system with higher comfort had no effect [8,11,17] on the feeding frequency compared to concrete slatted floors. The eating behavior and rumination depend more on the animal size, feeding management and chemical and physical properties of the feed [24], as is the case in dairy cattle. All of the above factors were fixed in the present study and therefore did not differ between groups. When feeding is noncompetitive, i.e., the space in the pen and at the feeding manger are not limited, differences between animals are not expected [24]. It is well known that there are differences in the eating behavior among breeds. A study comparing Jersey and Holstein cows found that there were no differences in eating time, but Holstein cows ruminate for a longer period of time [25]. In the present trial, we did not observe any difference in the eating behavior and rumination between breeds, probably due to the absence of differences in size and other physiological differences between the breeds used, as suggested in the latter trial.

Animal welfare is positive when animals show active and positive interaction with the environment and other animals in the group. This leads to exploration, foraging, hunting, bonding and social contact [26]. Animals exhibit these behaviors in situations where they are not exposed to threats or harmful environments [26]. In the present study, the comparison between HWF and CSF showed that animals on HWF showed higher frequencies of grooming, rubbing and aggression but not stereotypy or mounting. The observed behaviors may indicate that heifers housed on HWF felt more comfortable and secure expressing these behaviors than those on CSF. Although fighting/aggression is associated with bad welfare, play fighting is a sign of good welfare [27]. This is partially consistent with research on Charolais and Limousin fattening bulls, where bulls housed on a rubber-covered slatted floor showed a higher number of head/butt displacement and chasings than those housed on a concrete slatted floor [17]. In addition, Limousin bulls had a higher number of mountings on a rubber-covered slatted floor than on a noncovered slatted floor [17]. On the other hand, there were no differences in the social interactions between beef cattle kept on deep litter and a fully slatted floor [11,23], and between beef cattle kept on concrete and a rubber floor [10]. To the best of our knowledge, there are no studies that have examined the social behavior of cattle housed on HWF. This might actually be better for animal welfare than deep litter or rubber-covered slatted floors because the floor is covered with a plastic mesh and therefore is not as slippery as rubber, but these assumptions should be investigated further.

Cattle prefer soft bedding, such as straw or soft rubber, to concrete or sandy materials [28,29]. Cattle also prefer dry surfaces to wet surfaces or surfaces covered with slurry, especially when lying down [30] and also walking [31]. Furthermore, if the ground on which the cattle lie is wet or muddy, this limits the animals area exposed to the floor, and, although they lie for less time in muddy conditions, the cleanliness score is lower [32]. The drainage system and the floor type are the most important factors affecting the cleanliness of the animals [33]. Therefore, we expected that cleanliness scores would be higher for cattle on HWF than on CSF due to rapid drainage of urine and water. In the present trial, animals on HWF appeared cleaner than those on CSF in all the observed body areas, except at the tail head. This is partially in correlation with other studies showing that cattle are significantly cleaner on straw than on slatted or rubber floors [12]. On the other hand, bulls reared on deep litter [11,23] or rubber floors were dirtier [15,17] than those reared on fully slatted floors. Results support the hypothesis that the rapid drainage and the material of the HWF could improve the cleanliness of the animals in this trial, and be better from a hygienic point of view than deep litter and rubber-covered floors. Moreover, heavier animals tend to lay more, and are therefore dirtier, as has been shown before in bulls [23]. This was also shown in the trial comparing Charolais and Limousin bulls on a concrete or a rubber-covered slatted floor, where Charolais bulls tended to be dirtier [17]. This finding is in line with the present trial: Charolais heifers tended to be dirtier than Limousins. These results could be due to the lower mass of Limousin heifers at the end of the trial and the slightly lower laying time than that of Charolais heifers.

Due to the design of the study, the presented results have some minor limitations since the pens within each group were not duplicated. Therefore, we switched the animals after two observations in order to gain some statistical relevance. Further investigation after this case study is suggested.

## 5. Conclusions

In the present study, the high welfare floor appeared to have no effect on the main performance parameters, standing, laying and eating behavior, compared to the concrete slatted floor. On the other hand, it seems that grooming, rubbing and aggression occurred more frequently in animals housed on the high welfare floor, which could indicate a higher welfare level and more species-specific behavior of animals housed on this floor system. In addition, animals housed on the high welfare floor appeared to be cleaner than those housed on a concrete slatted floor, which could also improve some health problems and animal welfare.

## Figures and Tables

**Table 1:** Weight (kg) and weight gain (kg/day) during the trial in heifers of Charolais and Limousin breed housed in high welfare floor or concrete slatted floor (means ± std).

Part	Consecutive Weighing	HWF		CSF	
		CHA	LIM	CHA	LIM
1	1	374 ± 41.5	333 ± 24.1	361 ± 26.9	335 ± 32.7
	2	410 ± 46.2	358 ± 24.2	395 ± 32.2	361 ± 31.0
	3	438 ± 48.1	380 ± 22.2	420 ± 34.0	384 ± 31.6
Switch					
2	4	420 ± 34.0	384 ± 31.6	438 ± 48.1	380 ± 22.2
	5	441 ± 32.1	404 ± 31.2	458 ± 47.4	399 ± 22.2
	6	464 ± 33.6	422 ± 33.0	479 ± 49.1	416 ± 20.5
	Weight gain				
1	(kg/day)	1.14 ± 0.20	0.82 ± 0.10	1.06 ± 0.20	0.88 ± 0.08
Switch					
2		0.78 ± 0.11	0.68 ± 0.08	0.73 ± 0.07	0.65 ± 0.06

High welfare floor (HWF), concrete slatted floor (CSF), Charolais (CHA), Limousin (LIM).

**Table 2:** Behavioral traits of the Charolais and Limousin heifers reared on high welfare floor or concrete slatted floor (M = means ± std).

Behaviour (%)	HWF		CSF	
	CHA	LIM	CHA	LIM
Standing	76.1 ± 25.4	79.4 ± 22.7	73.0 ± 25.5	77.2 ± 20.4
Lying	24.0 ± 25.4	20.6 ± 22.7	27.0 ± 25.5	22.8 ± 20.4
Inactive—standing	22.6 ± 11.0	27.0 ± 13.7	22.2 ± 15.8	23.6 ± 12.0
Inactive—lying	7.4 ± 10.5	7.6 ± 11.4	8.62 ± 9.6	7.57 ± 8.70
Eating	40.2 ± 24.0	39.1 ± 24.1	40.1 ± 27.6	43.8 ± 25.5
Rumination	22.6 ± 17.5	21.4 ± 18.2	25.2 ± 19.2	20.0 ± 15.1
Stereotypy	0.298 ± 1.684	0.000 ± 0.000	0.037 ± 0.210	0.074 ± 0.293
Grooming	5.10 ± 3.52	3.12 ± 2.91	2.86 ± 2.35	3.54 ± 2.66
Rubbing	1.83 ± 2.06	1.89 ± 2.15	0.93 ± 1.41	1.35 ± 1.74
Occurrences/h				
Drinking	4.38 ± 4.40	4.34 ± 2.61	3.69 ± 2.93	4.633.32
Aggression	2.69 ± 3.01	3.59 ± 4.54	1.75 ± 2.26	1.41 ± 1.36
Mounting	0.25 ± 0.51	0.19 ± 0.47	0.12 ± 0.42	0.22 ± 0.55

High welfare floor (HWF), concrete slatted floor (CSF), Charolais (CHA), Limousin (LIM).

**Table 3:** Cleanliness of the Charolais and Limousin heifers reared on high welfare floor or concrete slatted floor (means ± std).

Body Part (Score)	HWF		CSF	
	CHA	LIM	CHA	LIM
Tail head	1.57 + 0.51	1.71 + 1.07	1.86 + 0.77	1.71 + 0.61
Upper rear limb	3.14 + 0.77	2.36 + 1.01	3.64 + 0.84	4.07 + 0.73
Ventral abdomen	3.21 + 0.89	2.21 + 0.70	3.71 + 0.99	3.57 + 0.85
Lower front limb	3.86 + 0.53	2.36 + 1.01	4.00 + 0.68	3.64 + 0.84
Lower rear limb	2.86 + 0.66	2.28 + 0.82	3.50 + 1.09	3.36 + 0.84
∑	14.64 + 2.06	10.93 + 3.71	16.71 + 3.27	16.36 + 2.47

High welfare floor (HWF), concrete slatted floor (CSF), Charolais (CHA), Limousin (LIM).

## Data Availability

The data presented in this study is available on reasonable request from the corresponding author.
